# Idelalisib treatment prior to allogeneic stem cell transplantation for patients with chronic lymphocytic leukemia: a report from the EBMT chronic malignancies working party

**DOI:** 10.1038/s41409-020-01069-w

**Published:** 2020-10-02

**Authors:** Johannes Schetelig, Patrice Chevallier, Michel van Gelder, Jennifer Hoek, Olivier Hermine, Ronjon Chakraverty, Paul Browne, Noel Milpied, Michele Malagola, Gerard Socié, Julio Delgado, Eric Deconinck, Ghandi Damaj, Sebastian Maury, Dietrich Beelen, Stéphanie Nguyen Quoc, Paneesha Shankara, Arne Brecht, Jiri Mayer, Mathilde Hunault-Berger, Jörg Bittenbring, Catherine Thieblemont, Stéphane Lepretre, Henning Baldauf, Liesbeth C. de Wreede, Olivier Tournilhac, Ibrahim Yakoub-Agha, Nicolaus Kröger, Peter Dreger

**Affiliations:** 1 Medical Clinic I, University Hospital, Dresden, Germany; 2 DKMS, Dresden, Germany; 3 CHU Nantes, Nantes, France; 4 University Hospital Maastricht, Maastricht, The Netherlands; 5 EBMT Data Office, Leiden, The Netherlands; 6 Department of Hematology, Necker Hospital and INSERM U1163 Imagine Institute, University of Paris, Paris, France; 7 Cancer Institute and Institute of Immunity and Transplantation, University College London Hospital, London, UK; 8 Hope Directorate, Dublin, Ireland; 9 CHU Bordeaux, Pessac, France; 10 Bone Marrow Transplant Unit, ASST-Spedali Civili di Brescia, University of Brescia, Brescia, Italy; 11 Hopital St. Louis, Paris, France; 12 Hospital Clinic, Barcelona, Spain; 13 Hopital Jean Minjoz, Besancon, France; 14 Centre Hospitalier-Universitaire, Institut d’Hématologie, Normandie University, Caen, France; 15 Service d’Hématologie Clinique et de Thérapie Cellulaire Creteil, CHU Henri Mondor, Créteil, France; 16 University Hospital, Essen, Germany; 17 Hopital la Pitié-Salpêtrière, Universite Paris IV, Paris, France; 18 Birmingham Heartlands Hospital, Birmingham, UK; 19 Helios Dr. Horst Schmidt Kliniken, Wiesbaden, Germany; 20 University Hospital Brno, Brno, Czech Republic; 21 Maladies du Sang, CHU Angers, Angers, France; 22 University of Saarland, Homburg, Saar Germany; 23 Hôpital St. Louis, Paris, France; 24 Inserm U1245 and Department of Hematology, Centre Henri Becquerel, Normandie University, Rouen, France; 25 Department of Medical Statistics & Bioinformatics, Leiden University Medical Center, Leiden, The Netherlands; 26 Service Therapie Cellulaire & Hematologie Cliniquer, Centre Hospitalier Universitaire, Clermont Ferrand, France; 27 Centre Hospitalier Universitaire de Lille, LIRIC, INSERM U995, Université de Lille, Lille, France; 28 University Hospital Eppendorf, Hamburg, Germany; 29 University of Heidelberg, Heidelberg, Germany

**Keywords:** Chronic lymphocytic leukaemia, Stem-cell therapies, Immunotherapy

## Abstract

No studies have been reported so far on bridging treatment with idelalisib for patients with chronic lymphocytic leukemia (CLL) prior to allogeneic hematopoietic cell transplantation (alloHCT). To study potential carry-over effects of idelalisib and to assess the impact of pathway-inhibitor (PI) failure we performed a retrospective EBMT registry-based study. Patients with CLL who had a history of idelalisib treatment and received a first alloHCT between 2015 and 2017 were eligible. Data on 72 patients (median age 58 years) were analyzed. Forty percent of patients had *TP53*_mut/del_ CLL and 64% had failed on at least one PI. No primary graft failure occurred. Cumulative incidences of acute GVHD °II–IV and chronic GVHD were 51% and 39%, respectively. Estimates for 2-year overall survival (OS), progression-free survival (PFS), and cumulative incidences of relapse/progression (CIR) and non-relapse mortality NRM were 59%, 44%, 25%, and 31%. In univariate analysis, drug sensitivity was a strong risk factor. For patients who had failed neither PI treatment nor chemoimmunotherapy (CIT) the corresponding 2-year estimates were 73%, 65%, 15%, and 20%, respectively. In conclusion, idelalisib may be considered as an option for bridging therapy prior to alloHCT. Owing to the high risk for acute GVHD intensified clinical monitoring is warranted.

## Introduction

Pathway inhibitors (PI) such as BTK-inhibitors (BTKi), PI3-Kinase inhibitors (PI3Ki), and BCL2-inhibitors (BCL2i) have fundamentally transformed the standard treatment landscape for chronic lymphocytic leukemia (CLL) in recent years. Since these drugs target different signaling pathways, CLL is usually not cross-resistant to different PIs. PIs can thus be administered sequentially [1–4]. Multiple sequences are perceivable in which BTKi and BCL2i are favored for first- and second line treatment [5–7].

The phosphatidylinositol 3-kinase (PI3Kδ)-inhibitor, idelalisib, can be active also in BTKi-refractory and BCL2i-refractory CLL [1, 5, 7], and thus might be an option for bridging into allogeneic hematopietic cell transplantation (alloHCT). The PI3-kinase exerts pleiotropic effects on cell metabolism, migration, proliferation, survival, and differentiation in lymphoid tissues. Autoimmune-mediated side effects such as colitis, hepatitis, and pneumonitis have been reported on treatment with idelalisib [8, 9]. Exposure to idelalisib could thus interfere with subsequent alloHCT. Patients who had received idelalisib for CLL prior to alloHCT have not been studied systematically so far.

Therefore, we analyzed the outcome of patients with CLL who had received idelalisib prior to alloHCT. In order to evaluate potential carry-over effects of idelalisib pretreatment on outcome after alloHCT we focused on early events after alloHCT such as engraftment, occurrence of GVHD and relapse as well as NRM throughout the first year after alloHCT. Here, we report final results from this EBMT registry study.

## Patients and methods

### Study design and patient eligibility

The study was designed as a registry-based retrospective multicenter study. Eligibility criteria were age 18 years or above, a first alloHCT for CLL between January 2015 and December 2017, and treatment with idelalisib at any time before transplant. Patients with a history of Richter transformation or syngeneic transplantation were excluded.

Since specific drug exposure was not routinely reported on Minimal Essential Data A (MED-A) forms during the beginning of this study, a survey was sent to all EBMT centers performing alloHCT in order to identify eligible patients. Baseline patient, disease, and transplant data of consecutive patients who were indicated by participating centers as meeting the eligibility criteria for this study were collected from MED-A forms.

The study was conducted by the EBMT Chronic Malignancies and Lymphoma Working Parties. All patients signed informed consent for the collection and registry-based analysis of their medical data. The study was conducted in full compliance with the declaration of Helsinki.

### Data management

For registered patients, baseline information, characteristics of the transplant procedure, and outcome data were collected on standard MED-A forms of EBMT. CLL with multiple cytogenetic abnormalities was classified according to the standard hierarchical approach [10]. Sensitivity of CLL to chemoimmunotherapy (CIT) was grouped in a modified way of the original definitions [11]. Patients whose CLL was responsive to CIT and did not require re-treatment for relapse within 2 years were classed as having CIT-sensitive CLL. Refractoriness to CIT or re-treatment within 2 years was classed as CIT-refractory CLL. Dose-intensity of the conditioning regimen was classified according to consensus working definitions of EBMT and CIBMTR [12].

### Statistical analysis

The primary aim of the analysis was to describe engraftment, GVHD, and events during the first year after alloHCT. Events for progression-free survival (PFS) were clinical relapse, progression, or death. Immune manipulations such as the taper of immunosuppressive drugs, the administration of donor lymphocyte infusions or the administration of rituximab were not considered as events for RFS. Non-relapse mortality (NRM) was defined as death without preceding clinical relapse/progression after alloHCT.

Data were analyzed as of September 20, 2019. Curves for RFS and overall survival (OS) were calculated using the Kaplan–Meier method and compared by log-rank tests. Incidences of relapse and NRM were calculated using cumulative incidence statistics and between-group comparisons were performed with the Gray test [13]. The impact of the occurrence GVHD (acute GVHD grades II to IV or any grade of chronic GVHD) on the incidence of relapse was tested in a time-dependent Cox-regression model. All point estimates for time-to-event endpoints are reported together with approximate 95%-confidence intervals.

## Results

### Patient characteristics

Altogether 72 patients met the eligibility criteria and had a full dataset available for this study.

Patient characteristics are shown in Table 1. Fifty-one male and 21 female patients were enrolled. The median age was 58 years (range, 36–73 years). Karnofsky Performance Score ranged between 80% and 100% for 98% of patients. The median reported Hematopoietic Cell Transplantation—Comorbidity Index was 0 (range: 0–6) but 16% of patients had a score ≥3.

**Table 1:** Patient characteristics.

	Numbers of patients (%) Total, *N* = 72
Median age at HCT [years] (range)	58 (36–73)
Patients older than 60 years	27 (38)
Female	21 (29)
Male	51 (71)
Karnofsky index at HCT	
100%	29 (44)
90%	22 (33)
80%	14 (21)
70%	1 (2)
Median HCT-CI (range)^a^	0 (0–6)
Patients with HCT-CI ≥3	8 (11)
Cytogenetic abnormalities	
* TP53*_del/mut_	29 (40)
Deletion(11q)/no *TP53*_del/mut_	7 (10)
Other/no del(11q), no *TP53*_del/mut_	13 (18)
None	23 (32)
Previous lines of therapies, median (range)	3 (1–8)
Idelalisib	
Median duration in months (range)	6 (1–28)
Idelalisib as last line prior to HCT	48 (67)
Idelalisib during course of CLL but not as last line	24 (33)
Drug exposure for CLL treatment, *N* of patients	
Chemotherapy naive	20 (28)
Purine analogue therapy	41 (57)
Ibrutinib	31 (43)
Venetoclax	13 (18)
Alemtuzumab	10 (14)
Chemoimmunotherapy sensitivity	
Not exposed	19 (26)
Sensitive disease	33 (45)
Poorly responsive/refractory^b^	20 (27)
Failure of at least one pathway inhibitor	46 (64)
Status at HCT	
Complete remission	5 (7)
Partial remission	54 (77)
Stable or progressive disease	11 (15)
Donor type	
HLA-identical sibling	21 (29)
Other (partially) matched related donor	10 (14)
8/8 HLA-compatible unrelated donor (UD)	25 (35)
HLA-compatible UD, HLA data missing	12 (17)
Partially matched UD	4 (6)
CMV constellation	
Donor and recipient CMV neg.	19 (27)
Donor or recipient CMV pos.	52 (73)
Sex constellation	
Female patient–female donor	10 (14)
Female patient–male donor	11 (16)
Male patient–female donor	14 (20)
Male patient–male donor	35 (50)
Conditioning regimen	
Non-myeloablative based on 2 Gray TBI	11 (15)
Reduced intensity	47 (65)
High-dose therapy	14 (19)
Stem cell source	
Peripheral blood stem cells	67 (93)
Bone marrow	4 (6)
Cord blood	1 (1)
GVHD prophylaxis	
CSA with/without MTX/MMF	62 (86)
Tacrolimus with/without MTX/MMF	8 (11)
Other	2 (3)
ATG	39 (54)
PTCY	9 (13)
Alemtuzumab	9 (13)

*N* number, *HCT* hematopoietic cell transplantation, *HCT-CI* hematopoietic cell transplantation—comorbidity index, *CMV* cytomegalovirus, *GVHD* graft-versus-host disease, *TBI* total body irradiation, *CSA* cyclosporine A, *MTX* methotrexate, *MMF* mycophenolate mofetil, *PBSC* peripheral blood stem cells, *ATG* anti-thymocyte globulin, *PTCY* posttransplant cyclophosphamide.

^a^Information on the HCT-CI was not available for 22 patients.

^b^No response or re-treatment within 24 months.

The median interval between diagnosis of CLL and alloHCT was 7 years (range, 1–19 years), and the median time from first treatment of CLL to alloHCT was 51 months (range, 4 months to 17 years). Twenty-nine patients (40%) had a deletion *TP53* mutation or deletion, seven patients (10%) had a deletion(11q) but no *TP53* mutation or deletion.

### Pre-treatment

By definition, all 72 patients had been exposed to idelalisib prior to alloHCT. Of those, 48 patients (67%) had received idelalisib as bridge to transplant. Twenty-two (46%) of these 48 patients had failed already at least one other PI. The interval between the last dose of idelalisib and alloHCT ranged between a minimum of 6 days and a maximum of 17 months with a median number of 24 days. In total, 16 patients had received their last dose of idelalisib within the last month prior to alloHCT. The response rate prior to alloHCT was 75% (36 out of 48 patients) among patients whose last line of treatment contained idelalisib. Regarding disease status prior to conditioning of all 72 patients, five patients (7%) were in complete remission, 54 patients (77%) were in partial remission, and 11 patients had stable/progressive CLL. The remission status was unknown for two patients.

Overall, the majority of patients had advanced disease stages reflected by a median number of 3 (range, 1–8) lines of therapy prior to alloHCT. Forty-five percent of patients had CIT-refractory disease. As for PI treatment, 43% had been exposed to ibrutinib and 18% to venetoclax during their course of CLL. Altogether, 64% of patients had failed one or more PIs. Eight patients (11%) had received three PIs (idelalisib, ibrutinib, and venetoclax) prior to alloHCT.

Of note, 20 patients (27%) had received only PI with or without monoclonal antibodies but no chemotherapy for the treatment of CLL. This subset exposed high-risk genetic features, with 75% of patients having *TP53*_mut/del_ CLL and 45% having failed at least on one PI, so that the median number of pre-treatments was still 2 (range, 1–4 lines of pretreatment).

### Transplant procedure

Forty-seven patients (65%) received reduced-intensity conditioning based on combinations of fludarabine and either busulphan, melphalan, or cyclophosphamide or total body irradiation (TBI) at cumulative doses between 2 and 8 Grays. Eleven patients (15%) received non-myeloablative conditioning based on TBI with 2 Gray and 14 patients (19%) received myeloablative conditioning.

Twenty-one patients (29%) received hematopoietic stem cells from their HLA-compatible siblings. Ten patients (14%) had other related donors (partially matched/haploidentical) and 41 patients (57%) had HLA-compatible unrelated donors, including four patients whose donors had a single HLA mismatch at HLA-A, -B, -C, or HLA-DRB1. The exact HLA-matching status was missing for 12 unrelated patient–donor pairs.

G-CSF mobilized peripheral blood stem cells were transplanted to 67 patients (93%), four patients received bone marrow and one patient cord blood. No patient received an in vitro T-cell depleted stem cell graft. GVHD prophylaxis was based on calcineurin inhibitor for 71 patients (97%). In addition 39 patients (54%) had received anti-thymocyte globulin (ATG), and nine patients (13%) each alemtuzumab or high-dose cyclophosphamide, respectively.

### Engraftment and GVHD

All patients showed primary engraftment. The median time to neutrophil engraftment was 17 days (range, 9–40 days). For platelet engraftment to greater than 20 GPt/L a median number of 16 days (range, 1–50 days) was reported. Two cases of secondary graft failure were observed in the context of continuous progression and a lethal infection in one patient each. Transient graft failure was reported for two patients. Idelalisib was the last line of treatment only in two of these four cases of secondary graft failure.

The cumulative incidences of acute GVHD grades II–IV and grades III–IV at 100 days after alloHCT were 51% (95%-CI, 39–63%) and 24% (95%-CI, 13–34%), respectively. The cumulative incidence of acute GVHD grades II–IV was 63% (95%-CI, 37–88%) among 16 patients whose last dose of idelalisib was within 28 days prior to alloHCT. Seventy patients were evaluable for chronic GVHD. The cumulative incidence of limited or extensive chronic GVHD at 1 year after alloHCT was 39% (95%-CI, 27–51%).

### Overall survival, progression-free survival, relapse, and non-relapse mortality

**Fig. 1:**
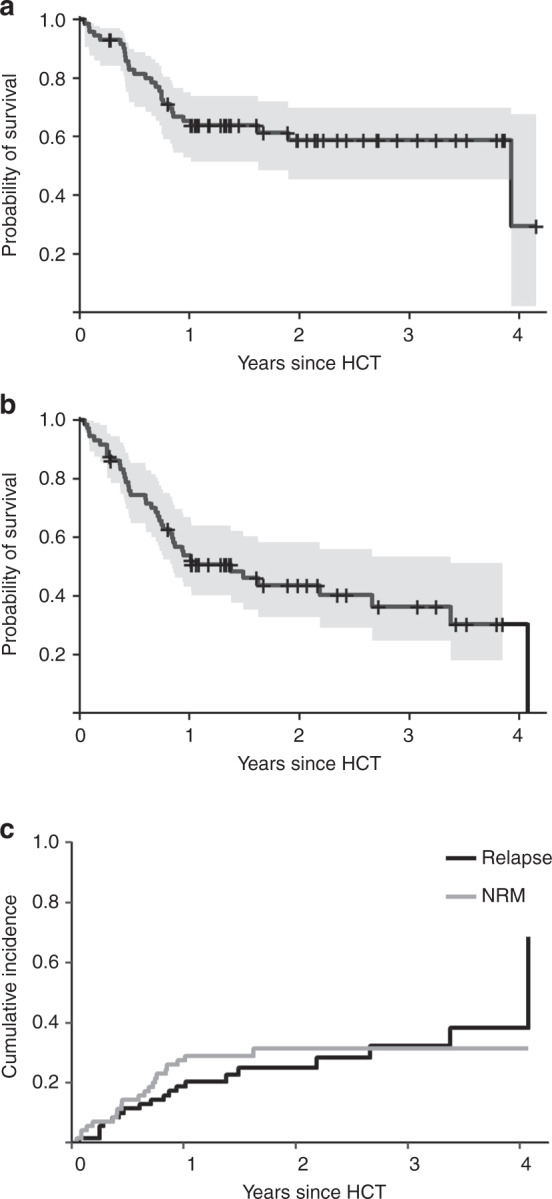
Outcome after alloHCT for patients with CLL who have been exposed to idelalisib. **a**, **b** Shows overall and progression-free survival with point-wise 95%-confidence intervals, respectively. **c** Shows the cumulative incidences of relapse and non-relapse mortality.

At last follow-up, 44 patients were alive with a median observation time of 21 months (range, 3–50 months). The probability of overall and PFS at 2 years was 59% (95%-CI, 45–70%) and 44% (95%-CI, 33–58%), respectively (Fig. 1a, b). Altogether 20 patients were reported to have experienced relapse or progression. The cumulative incidence of relapse at 2 years was 25% (95%-CI, 14–36%) (Fig. 1c). After relapse/progression, ibrutinib was administered to seven patients, venetoclax to four patients, and idelalisib to one patient. One patient received all three drugs after having experienced relapse but died 47 months after alloHCT. Two patients received venetoclax after alloHCT without previous hematologic relapse/progression as maintenance therapy.

Twenty-one patients died without previous relapse. Main causes of death were infections (*N* = 8), GVHD (*n* = 7), organ failure (*N* = 2), and a secondary malignancy (*N* = 1). Causes of death were not reported for three patients. The cumulative incidence of NRM at 2 years was 31% (95%-CI, 20–43%) (Fig. 1c). Notably, no patient whose last dose of idelalisib was within 28 days prior to alloHCT died within the first 100 days after alloHCT.

### Univariable comparisons

In exploratory univariable analyses, age, HCT-CI, number of prior treatment lines, remission status, and donor type did not have a significant impact on OS, PFS, NRM, and CIR (see Table 2).

**Table 2:** Univariable analysis of overall- and relapse-free survival, relapse incidence and non-relapse mortality.

Variables	*N*	2-year OS (95% CI)	*p* value	2-year PFS (95% CI)	*p* value	2-year CIR (95% CI)	*p* value	2-year NRM (95% CI)	*p* value
Whole cohort	72	59 (45–70)		44 (33–58)		25 (14–36)		31 (20–43)	
Patient age at HCT									
<60 years	45	66 (52–83)	0.13	52 (39–70)	0.13	24 (11–38)	0.62	23 (10–36)	0.11
≥60 years	27	47 (30–74)		28 (14–58)		26 (6–46)		45 (23–68)	
HCT-CI, score									
<3	42	52 (36–73)	0.45	40 (26–61)	0.49	22 (8–36)	0.72	38 (21–55)	0.54
≥3	8	50 (25–100)		38 (15–92)		25 (0–58)		38 (0–75)	
Presence of *TP53*_mut/del_ CLL									
Present	29	86 (73–100)	0.001	68 (51–90)	0.002	25 (6–44)	0.46	7 (0–17)	0.001
Absent	43	42 (29–61)		28 (17–47)		25 (11–39)		47 (31–63)	
Status with respect to chemotherapy									
Chemo-naive CLL	20	73 (55–96)	0.16	64 (44–94)	0.02	14 (0–34)	0.06	21 (2–41)	0.16
Chemo-exposed CLL	51	52 (39–70)		38 (26–55)		27 (14–39)		36 (22–50)	
Status with respect to PI									
No PI failed, Idela sensitive	26	76 (61–95)	0.06	67 (50–89)	0.01	17 (1–34)	0.13	16 (1–31)	0.03
≥1 PI failed	46	48 (33–68)		30 (18–50)		30 (15–45)		41 (25–57)	
Chemoimmunotherapy sensitivity									
Not exposed	20	73 (55–96)	0.13	64 (44–94)	0.03	14 (0–34)	0.12	21 (2–41)	0.22
Sensitive disease	18	60 (38–95)		50 (32–79)		22 (2–42)		28 (6–49)	
poorly responsive/refractory	33	47 (32–70)		32 (19–54)		29 (12–45)		39 (21–58)	
Treatment failure status									
Failed neither PI nor CIT	16	73 (54–100)	0.003	64 (43–96)	<0.001	15 (0–37)	0.047	20 (0–41)	0.005
Failed PI either or CIT	32	68 (52–90)		59 (44–79)		19 (5–33)		22 (7–37)	
Failed PI and CIT	23	31 (16–62)		13 (4–43)		33 (12–54)		54 (30–78)	
Number of lines of pretreatment									
≤2	23	68 (51–91)	0.37	56 (38–83)	0.13	17 (0–35)	0.16	27 (8–47)	0.45
≥3	49	54 (40–72)		38 (26–56)		28 (15–41)		34 (19–48)	
Remission status at alloHCT									
Complete/partial remission	59	62 (50–78)	0.09	45 (33–62)	0.32	27 (14–40)	0.57	28 (15–40)	0.053
Stable/progressive disease	13	42 (22–82)		35 (16–76)		15 (0–36)		50 (19–81)	
Donor type									
HLA-identical sibling	21	58 (40–86)	0.68	50 (32–79)	0.38	19 (2–36)	0.39	31 (9–53)	0.70
Alternative donor	51	61 (48–76)		41 (28–59)		28 (14–42)		31 (18–44)	

*OS* overall survival, *RFS* relapse-free survival, *RI* relapse incidence, *NRM* non-relapse mortality, *CI* confidence interval, *HCT* hematopoietic stem cell transplantation, *HCT-CI* hematopoietic cell transplantation—comorbidity index, *p* values are based on log-rank test (OS and PFS) and Gray’s test (CIR and NRM), they compare the curves during the whole follow-up.

**Fig. 2:**
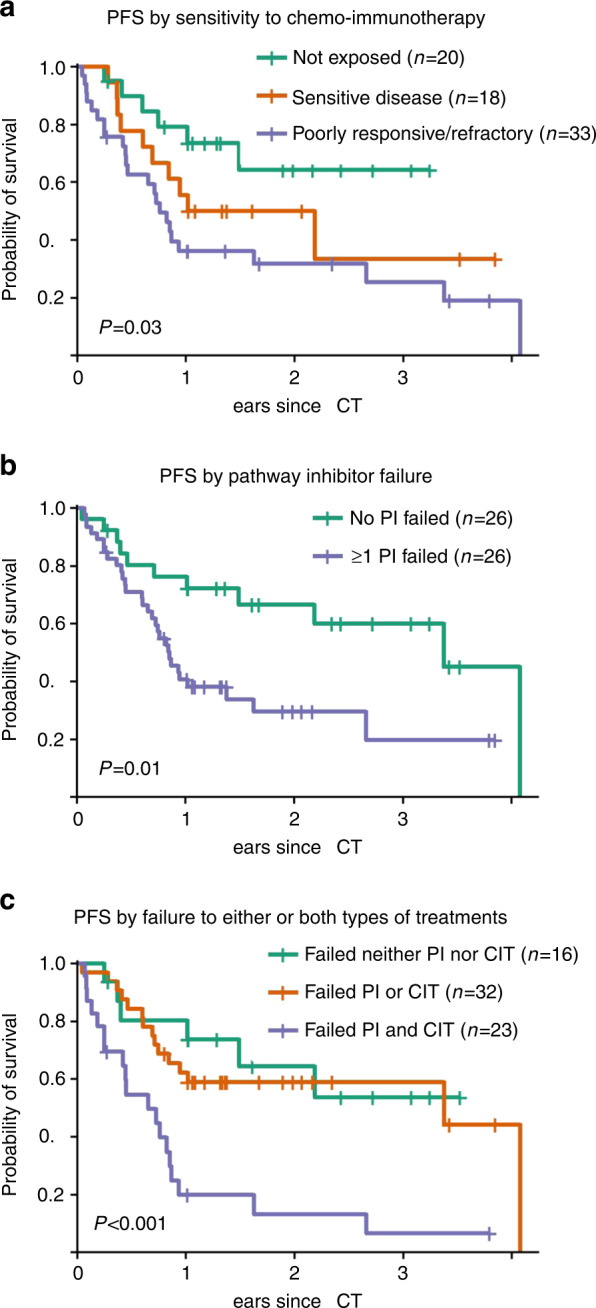
Univariable comparisons of progression-free survival for selected risk factors were done by log-rank tests. **a** Shows progression-free survival by sensitivity to chemoimmunotherapy (CIT). Patients who were not exposed to CIT are shown in green, patients with CIT-sensitive CLL in brown and patients with poorly responsive/refractory CLL in violet. **b** Shows PFS by pathway-inhibitor failure. Patients who had only been exposed to idelalisib and responsive disease are displayed in green, all remaining patients are displayed in violet. **c** Shows PFS of patients by pathway-inhibitor (PI)- or CIT failure. Patients who had failed neither PI nor CIT are shown in green. Patients who had failed either PI or CIT are shown in brown color. The remaining patients (violet curve), who have failed both, CIT and PI, have the worst outcome.

First, we analyzed PFS by grouping patients according to the type of pretreatment and disease sensitivity (see Fig. 2a–c). Sensitivity to CIT prior to conditioning had a significant impact on PFS in univariable comparison (log-rank test, *p* = 0.03). Patients with CIT-naive CLL had 2-year PFS of 64% (95%-CI, 39–89%), patients with CIT-sensitive CLL had 2-year PFS of 50% (95%-CI, 26–74%), and patients with CIT-refractory CLL had 32% (95%-CI, 14–50%) (Fig. 2a).

Patients who had PI-sensitive disease had better 2-year PFS compared to patients who had failed at least one PI (67% (95% CI, 47–87%) versus 30% (95% CI, 14–46%), log-rank test, *p* = 0.01) (Fig. 2b). Notably, owing to the design of this study all patients with PI-sensitive CLL were sensitive to idelalisib. In contrast, of eight patients who had been exposed to all three PIs (BTKi, BCL2i, and PI3K) and had failed on two PIs only two patients (25%) did not experience relapse (*N* = 3, 37.5%) or NRM (*N* = 3, 37.5%) during the first year after HCT.

Finally, we analyzed the impact of high-risk genetic abnormalities in this cohort of patients. Two-year PFS was 86% (95% CI, 73–100%) for patients with *TP53*_del/mut_ CLL versus compared to 28% (95% CI, 17–47%) for the remaining patients (Log-rank test, *p* = 0.002). Of note in this context, patients with *TP53*_del/mut_ more often had chemotherapy-naive CLL prior to the start of conditioning compared to patients without *TP53*_del/mut_ CLL (88% versus 48%, chi-square test *p* < 0.001) although the percentage of patients who had failed at least one PI was not significantly different among the two groups (55 versus 69%, respectively, chi-square test *p* = 0.2).

## Discussion

We present results of a large cohort of contemporary patients with who received alloHCT for high-risk CLL after pretreatment with idelalisib. This cohort of patients had two unique features: First, it is the largest cohort published so far of patients who failed on one or two PIs and subsequently proceeded to alloHCT. Second, it comprises a large number of patients whose CLL was chemotherapy naive at alloHCT. Results of this retrospective, EBMT registry-based study allow for a discussion of the risk-benefit ratio of idelalisib as a bridging treatment prior to alloHCT and—from a broader perspective—to reason about contemporary indications for alloHCT for CLL.

Safety of idelalisib treatment prior to alloHCT has been a concern due to the observation of autoimmune-mediated diseases, resulting in severe colitis, pneumonitis, and hepatitis, which may occur during idelalisib treatment [8]. Experimental data suggest that PI3K delta inhibitors, such as idelalisib, partly mediate their activity by disrupting the function of immunosuppressive cell populations such as *T*_regs_ and MDSCs [14]. Thereby idelalisib may shift the balance from immune tolerance toward effective antitumor immunity [15]. Idelalisib may thus exert direct and indirect action on tumor cells but also unleash autoimmune reactions. Potential carry-over effects of a pretreatment with idelalisib were therefore a valid concern. Overall, the cumulative incidences of 51% grades II–IV acute GVHD and 39% chronic GVHD observed here are comparable to recent publications on alloHCT for contemporary patients with CLL [16–18]. E.g. after pretreatment with ibrutinib cumulative incidences of 49% grades II–IV acute GVHD and 54% chronic GVHD were reported [17] and recently for patients who received conditioning with fludarabine and low-dose TBI combined with rituximab cumulative incidences of 69% grades II–IV acute GVHD and 66% chronic GVHD were published [18]. Still, the observation that 63% of patients experienced grades II–IV acute GVHD who had taken their last dose of idelalisib within 28 days to alloHCT indicates that this patient population is at high risk of GVHD owing to multiple factors, e.g., due to frequent partially mismatched related and unrelated transplantation.

Little information is available on the safety of idelalisib in the context of alloHCT. Sellner et al. reported EBMT registry data from 33 patients with follicular lymphoma who had received idelalisib for bridging to alloHCT [19]. They found no increased incidence of acute GVHD compared to patients who had received other treatment regimens prior to alloHCT but also reported a considerable incidence of severe acute GVHD (24% acute GVHD grades III–IV) in this patient population. On the other hand, Dreger et al. analyzed registry data on 24 patients with predominantly B-cell malignancies who had received idelalisib for the treatment of relapse after alloHCT and reported de novo GVHD or aggravation of preexisting GVHD in only one patient in whom idelalisib had been initiated as early as 30 days after alloHCT [20]. Taken together, currently available data are not robust enough to preclude an increased risk of GVHD after bridging therapy with idelalisib. Therefore, caution should be maintained when idelalisib is used in this vulnerable period, especially with respect to GVHD.

To our knowledge this is the largest cohort of contemporary patients after alloHCT of whom a large proportion had failed at least one PI and/or CIT. The heterogeneity of the pretransplant disease history allowed only for a preliminary univariable risk factor analysis. In this analysis, failure after CIT and/or on a PI was a significant risk factor for PFS. Data from a study on ibrutinib prior to alloHCT for CLL suggested already that PI failure might be a negative risk factor for relapse [17]. However, the observation that patients who had only failed PI treatment but not CIT had better outcomes inspite of *TP53*_mut/del_ CLL in 55% of cases (see Fig. 2c) than patients who had failed PI treatment and CIT inspite of only 17% of *TP53*_mut/del_ CLL warrants consideration.

Our observations fit to the common principle that results after alloHCT deteriorate the more lines of therapy have been administered prior to transplantation. So while alloHCT should not be delayed, we also would like to stress that the risk-benefit ratio of alloHCT compared to current standard treatment options, does not justify recommendation for alloHCT in first remission and refer to our consensus paper on indications for alloHCT in the era of PIs [21].

Once the decision was made to proceed to an allogeneic HCT, the most potent treatment should be chosen to induce a remission prior to starting conditioning. This recommendation is based on risk factor analyses from multiple studies, where the remission status prior to alloHCT turned out to be a major predictor for long-term disease free survival [22, 23]. Critical issues for this decision are the treatment history and the presence of mutations which confer resistance to CIT or certain PIs. Venetoclax may have the most attractive risk-benefit ratio for patients with ventoclax-naive or sensitive CLL, because it is well tolerated and MRD-negative responses have been reported even after failure of ibrutinib or idelalisib [2, 3, 5]. However, data on venetoclax-induction immediately prior to alloHCT have not been published yet. Data on the efficacy of idelalisib after failure of PI treatment are sparse. In one retrospective analysis, the overall response rate was 46% with no complete remission among 37 patients who had received idelalisib after ibrutinib failure [5]. Recently, data on 17 patients were reported who had been treated with PI3Ki after BTKi exposure and failure after venetoclax [7]. Of these patients 47% achieved a remission, but the median PFS was only 5 months. Based on these data idelalisib may be an option for remission induction prior to alloHCT in e.g., patients with *TP53*_mut/del_ CLL who failed or did not tolerate BTKi and venetoclax.

In conclusion, results from this retrospective study allow for the use of idelalisib for remission induction prior to alloHCT. However, since we cannot exclude immunologic carry-over effects, we recommend stopping idelalisib at least 1 month prior to alloHCT and intensified monitoring for symptoms of acute GVHD.

Our study showed poor outcomes after alloHCT for patients who had failed PIs and CIT during their CLL treatment course. This observation may be used as an argument not to delay alloHCT in fit patients with high-risk CLL who have failed treatment with PIs. Alternatively, for this group of patients enrollment onto clinical trials with new investigational agents or chimeric antigen receptor modified T cells or NK cells may be an attractive option.
