# Correction to: Idelalisib treatment prior to allogeneic stem cell transplantation for patients with chronic lymphocytic leukemia: a report from the EBMT chronic malignancies working party

**DOI:** 10.1038/s41409-021-01516-2

**Published:** 2021-11-12

**Authors:** Johannes Schetelig, Patrice Chevallier, Michel van Gelder, Jennifer Hoek, Olivier Hermine, Ronjon Chakraverty, Paul Browne, Noel Milpied, Michele Malagola, Gerard Socié, Julio Delgado, Eric Deconinck, Ghandi Damaj, Sebastian Maury, Dietrich Beelen, Stéphanie Nguyen Quoc, Paneesha Shankara, Arne Brecht, Jiri Mayer, Mathilde Hunault-Berger, Jörg Bittenbring, Catherine Thieblemont, Stéphane Lepretre, Henning Baldauf, Liesbeth C. de Wreede, Olivier Tournilhac, Ibrahim Yakoub-Agha, Nicolaus Kröger, Peter Dreger

**Affiliations:** 1 Medical Clinic I, University Hospital, Dresden, Germany; 2 DKMS, Dresden, Germany; 3 CHU Nantes, Nantes, France; 4 University Hospital Maastricht, Maastricht, The Netherlands; 5 EBMT Data Office, Leiden, The Netherlands; 6 Department of Hematology, Necker Hospital and INSERM U1163 Imagine Institute, University of Paris, Paris, France; 7 Cancer Institute and Institute of Immunity and Transplantation, University College London Hospital, London, UK; 8 Hope Directorate, Dublin, Ireland; 9 CHU Bordeaux, Pessac, France; 10 Bone Marrow Transplant Unit, ASST-Spedali Civili di Brescia, University of Brescia, Brescia, Italy; 11 Hopital St. Louis, Paris, France; 12 Hospital Clinic, Barcelona, Spain; 13 Hopital Jean Minjoz, Besancon, France; 14 Centre Hospitalier-Universitaire, Institut d’Hématologie, Normandie University, Caen, France; 15 Service d’Hématologie Clinique et de Thérapie Cellulaire Creteil, CHU Henri Mondor, Créteil, France; 16 University Hospital, Essen, Germany; 17 Hopital la Pitié-Salpêtrière, Universite Paris IV, Paris, France; 18 Birmingham Heartlands Hospital, Birmingham, UK; 19 Helios Dr. Horst Schmidt Kliniken, Wiesbaden, Germany; 20 University Hospital Brno, Brno, Czech Republic; 21 Maladies du Sang, CHU Angers, Angers, France; 22 University of Saarland, Homburg, Saar Germany; 23 Hôpital St. Louis, Paris, France; 24 Inserm U1245 and Department of Hematology, Centre Henri Becquerel, Normandie University, Rouen, France; 25 Department of Medical Statistics & Bioinformatics, Leiden University Medical Center, Leiden, The Netherlands; 26 Service Therapie Cellulaire & Hematologie Cliniquer, Centre Hospitalier Universitaire, Clermont Ferrand, France; 27 Centre Hospitalier Universitaire de Lille, LIRIC, INSERM U995, Université de Lille, Lille, France; 28 University Hospital Eppendorf, Hamburg, Germany; 29 University of Heidelberg, Heidelberg, Germany

**Keywords:** Chronic lymphocytic leukaemia, Stem-cell therapies, Immunotherapy

Correction to: *Bone Marrow Transplantation* 10.1038/s41409-020-01069-w, published online 02 Oct 2020

The Idelalisib treatment prior to allogeneic stem cell transplantation for patients with chronic lymphocytic leukemia: a report from the EBMT chronic malignancies working party, written by Johannes Schetelig, Patrice Chevallier, Michel van Gelder, Jennifer Hoek, Olivier Hermine, Ronjon Chakraverty, Paul Browne, Noel Milpied, Michele Malagola, Gerard Socié, Julio Delgado, Eric Deconinck, Ghandi Damaj, Sebastian Maury, Dietrich Beelen, Stéphanie Nguyen Quoc, Paneesha Shankara, Arne Brecht, Jiri Mayer, Mathilde Hunault-Berger, Jörg Bittenbring, Catherine Thieblemont, Stéphane Lepretre, Henning Baldauf, Liesbeth C. de Wreede, Olivier Tournilhac, Ibrahim Yakoub-Agha, Nicolaus Kröger, Peter Dreger was originally published Online First without Open Access. After publication in volume 56, issue 3, page 605–613 the author decided to opt for Open Choice and to make the article an Open Access publication. Therefore, the copyright of the article has been changed to © The Author(s) 2020 and the article is forthwith distributed under the terms of the Creative Commons Attribution 4.0 International License, which permits use, sharing, adaptation, distribution and reproduction in any medium or format, as long as you give appropriate credit to the original author(s) and the source, provide a link to the Creative Commons licence, and indicate if changes were made. The images or other third party material in this article are included in the article’s Creative Commons licence, unless indicated otherwise in a credit line to the material. If material is not included in the article’s Creative Commons licence and your intended use is not permitted by statutory regulation or exceeds the permitted use, you will need to obtain permission directly from the copyright holder. To view a copy of this licence, visit http://creativecommons.org/licenses/by/4.0. Open access funding enabled and organized by Projekt DEAL.

